# Structural analysis of peptide binding to integrins for cancer detection and treatment

**DOI:** 10.1007/s12551-023-01084-3

**Published:** 2023-07-28

**Authors:** Mauricio Urquiza, Daniela Benavides-Rubio, Silvia Jimenez-Camacho

**Affiliations:** Chemistry Department, Faculty of Sciences, Universidad Nacional de Colombia, Carrera 30# 45-03, Ciudad Universitaria, Bogotá, Colombia

**Keywords:** Integrins, Complexes, Structure, Binding, Peptides, Cancer

## Abstract

Integrins are cell receptors involved in several metabolic pathways often associated with cell proliferation. Some of these integrins are downregulated during human physical development, but when these integrins are overexpressed in adult humans, they can be associated with several diseases, such as cancer. Molecules that specifically bind to these integrins are useful for cancer detection, diagnosis, and treatment. This review focuses on the structures of integrin-peptidic ligand complexes to dissect how the binding occurs and the molecular basis of the specificity and affinity of these peptidic ligands. Understanding these interactions at the molecular level is fundamental to be able to design new peptides that are more specific and more sensitive to a particular integrin. The integrin complexes covered in this review are α5β1, αIIbβ3, αvβ3, αvβ6, and αvβ8, because the molecular structures of the complex have been experimentally determined and their presence on tumor cancer cells are associated with a poor prognosis, making them targets for cancer detection and treatment.

Integrins are α and β heterodimeric transmembrane receptors expressed on various cell types and tissues throughout the body, playing important roles in cell adhesion, migration, signaling, motility, proliferation, polarity, differentiation, and survival. Despite the similarities in amino acid sequences and structures, the 24 integrins (built with 18α- and 8β-subunits) have different ligand-binding specificities.

Changes in the expression pattern of certain integrins contribute to cancer development by promoting tumor cell survival, metastasis, invasion, and extravasation. This review will focus on integrins involved in cancer development, having the three-dimensional structure determined in complexes with a peptidic ligand (see also Table 1). Understanding the molecular basis of ligand affinity and specificity for these integrins is helpful in designing molecules for tumor detection and treatment with high affinity and specificity (Urquiza et al. 2020; Takada et al. 2007; Desgrosellier and Cheresh 2010; Barczyk et al. 2010; Slack et al. 2022).

**Table 1:** The α5β1, αIIbβ3, αvβ3, αvβ6, and αvβ8 integrins in different types of cancer and the peptides designed for detection and treatments

Integrin	Cancer type	Peptides
α5β1	Diffuse-type gastric carcinoma (DGC), ovarian	ATN-161 (Ac-PHSCN-NH2), cilengitide (Sui et al. 2018)
αIIbβ3	Melanoma	GRGDSP (Zhu et al. 2013)
αvβ3	Melanoma, breast, ovarian	RGD-4 C (ACDCRGDCFCG), KCRGDCFC, LXW7, LXZ2, IsoDGR, RWrNM y RWrNK, RGDF cilengitide. (Gu et al. 2023)
αvβ6	Skin, colon, breast, lung, oral mucosa, cervix, salivary gland, liver, ovary, endometrium, gastric cancer, and colorectal cancer	A20FMDV2 (NAVPNLRGDLQVLAQKVART), A20 (Brzozowska and Deshmukh 2022) GRGDLGRLKK (Dong et al. 2014)
αvβ8	Glioblastoma, squamous cell carcinomas, and pancreatic cancer	GRRGDLAT (Wang et al. 2019)

## Integrin α5β1

Drug-resistant ovarian cancers have significantly higher α5β1-integrin expression than partially sensitive or sensitive ovarian cancers (Hu and Gao 2012). Moreover, the α5-integrin is overexpressed in 9% of ovarian cancer patients, and they have a lower survival rate (Sawada et al. 2008). Additionally, integrin α5 plays a crucial role in mediating cancer cell-fibroblast interactions during peritoneal dissemination of diffuse-type gastric carcinoma (DGC), which is associated with poor prognosis. Monoclonal antibodies (mab) recognizing integrin α5β1 inhibit the adhesion of DGC cells to cancer-associated fibroblasts, reducing diffusive infiltration, frequent peritoneal dissemination, and massive fibrosis (Miyamoto et al. 2022). Moreover, high levels of integrin α5β1 and galectin-1 in stromal cells are associated with no-response to cisplatin-based neoadjuvant chemotherapy in squamous cervical cancer patients (Zhu et al. 2017).

In contrast, integrin α5β1 expression is frequently lost in colorectal cancer cells, which is associated with proliferation and tumorigenicity involving HER-2 signaling. In fact, integrin α5β1 re-expression in colon cancer cells abrogates their tumorigenicity by increasing lysosomal targeting of HER-2 (Kuwada et al. 2005). Furthermore, integrin α5β1 binding to the secreted protein Tubulointerstitial nephritis antigen-like 1 (Tinagl1), which also involves αvβ1 and epidermal growth factor receptor (EGFR), results in suppressing triple-negative breast cancer progression and metastasis (Shen et al. 2019; Trerotola et al. 2013). There are several antagonists of integrin α5β1 in clinical trials, and the most successful ones are as follows:

1. ATN-161 (Ac-PHSCN-NH2), an integrin α5β1 antagonist, inhibits tumor angiogenesis and metastasis in various tumor types. ATN-161 blocks integrin α5β1 interactions, strongly inhibiting nuclear factor-κB (NF-κB) activation and matrix metalloproteinase-2/9 expression (Sui et al. 2018)
2. Cilengitide (Merck KGaA), a cyclic RGD pentapeptide that blocks the RGD integrin binding site, greatly hinders angiogenesis, starving the tumor, and in specific cases, improving patient treatment efficacy and outcome
3. Volociximab, a high-affinity chimeric antibody against human α5β1-integrin, inhibits fibronectin binding and endothelial cell survival and proliferation. In a xenografted ovarian cancer model, volociximab significantly reduced tumor burden

**Fig. 1:**
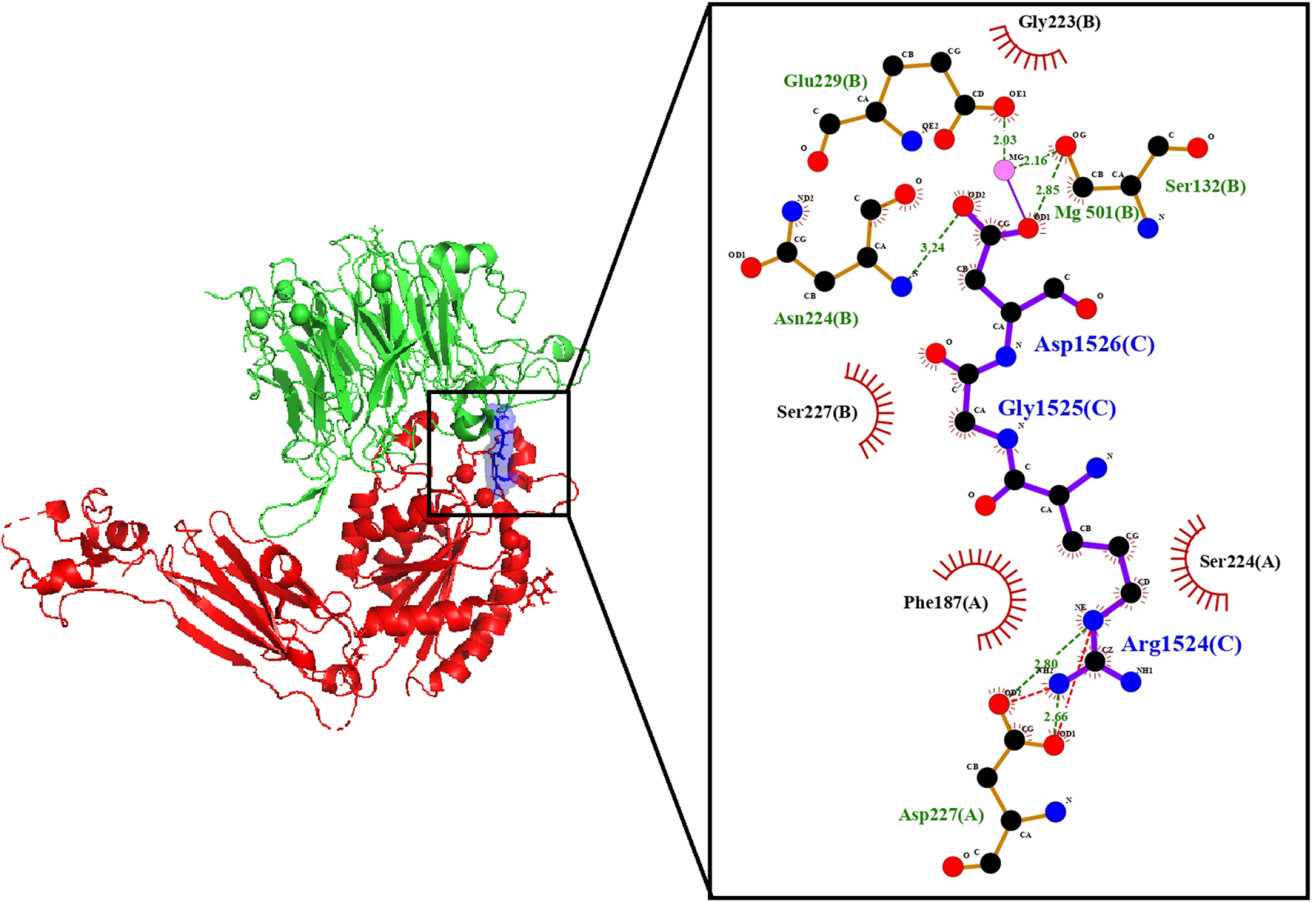
Integrin α5β1 in complex with RGD-peptide structure (α5-subunit in green, β1-subunit in red, and RGD peptide in blue) with its interaction map of the RGD-peptide (C) with the α5-subunit (A) and β-3 subunit (B)

The crystal structure of integrin α5β1 in complex with RGD-peptide at a resolution of 1.78 Å, downloaded from the Protein Data Bank (PDB: 4WK0)(Xia and Springer 2014), shows several water molecules between α5 and β1-subunits and magnesium and calcium ions (Fig. 1). These water molecules not only help to bind the two chains together but also to bind the RGD-peptide to both integrin subunits. The Arg-residue of RGD-peptide makes hydrogen bonds with two water molecules connected to F-187, S-224, and D-227 residues from α5-subunit. The Gly-residue of the RGD-peptide interacts with three water molecules, S-227 from β1-subunit, also F-187 from α5-subunit. The Asp-residue of the RGD-peptide interacts with four water molecules, two magnesium ions and N-224, and E-229 from β1-subunit. Additionally, it makes contact with a water molecule and D-227 from α5-subunit and a magnesium ion. Asp from RGD-peptide binds to a water molecule and to the β1-subunit residues G-223, and N-224 directly plus S-132, and E-229 through a magnesium ion.

### Integrin αIIbβ3

The integrin αIIbβ3 is the most abundant receptor on platelets, which binds to peptides and proteins containing the RGD sequence present on different adhesive proteins, including fibrinogen, VWF, fibronectin, and vitronectin. The integrin αIIbβ3 is also involved in cancer progression, tumor cell proliferation, metastasis, and promoting tumor cell adhesion and invasion. Additionally, αIIbβ3 facilitates interaction with tumor cells through binding with podoplanin, P-selectin glycoprotein ligand-1 (PSGL-1), A disintegrin and metalloproteinase domain-containing protein 9 (ADAM-9), and fibrinogen/αvβ3. This interaction probably creates a physical shield around cancer cells, protecting them from the immune system (Boukerche et al. 1989; Grossi et al. 1988; Honn et al. 1992; Timar et al. 1998; Nierodzik et al. 1992; Wagner et al. 1996; Mammadova-Bach et al. 2016; Tesfamariam 2016).

**Fig. 2:**
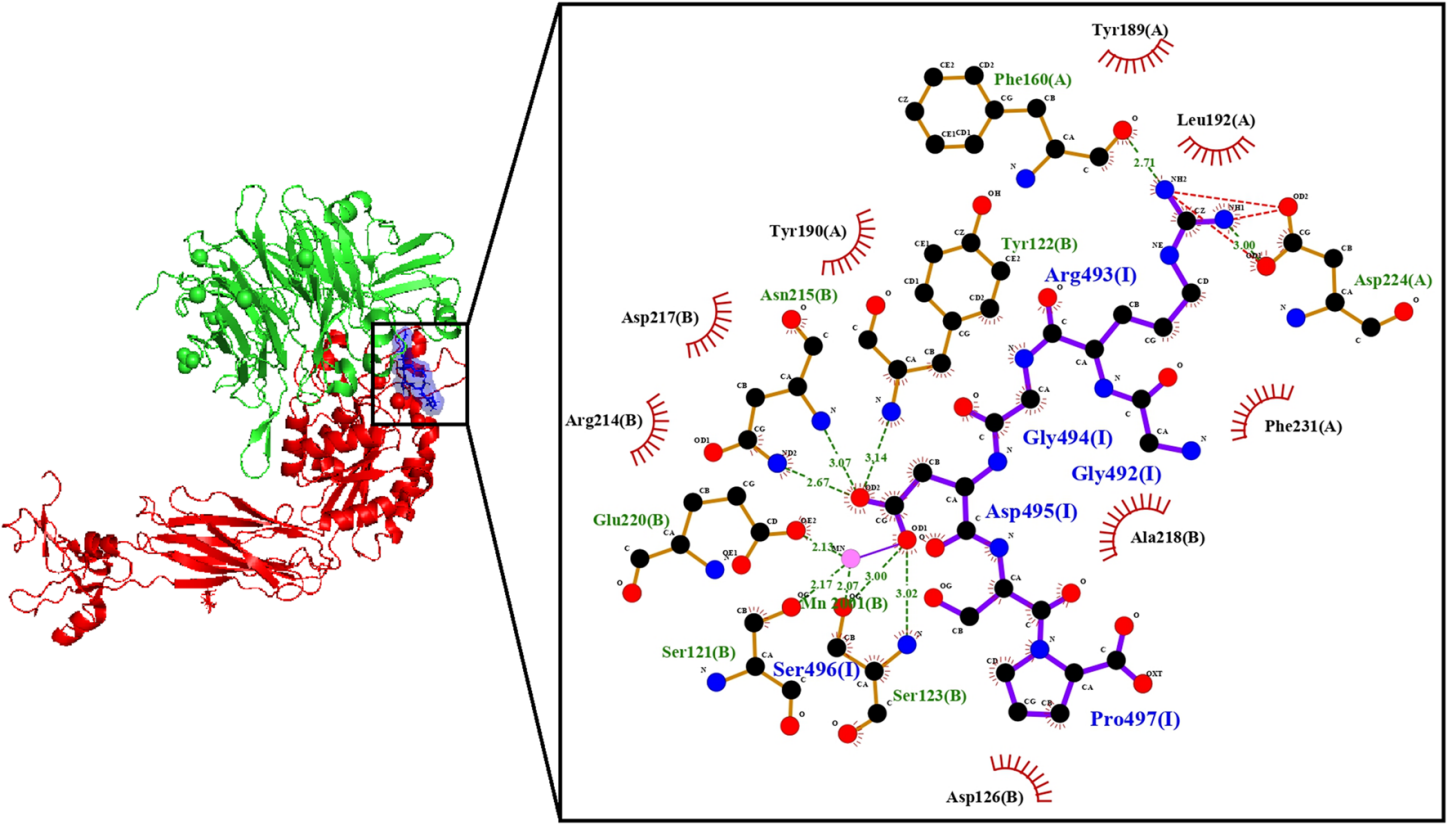
Integrin αIIBβ3 and RGD peptide complex (αIIb subunit in green, β3 subunit in red, and RGD peptide in blue) with its interaction map of the RGD-peptide (I) with the α5-subunit (A) and β-3 subunit (B)

The crystal structure of integrin αIIBβ3 in complex with GRGDSP peptide has been obtained at a resolution of 3.00 Å (PDB: 3ZE1) (Zhu et al. 2013) (Fig. 2). The structure reveals three manganese ions bound to the β3 subunit in close proximity to the GRGDSP peptide binding site, with one of them bound to S-121, S-123, and D-119. The Arg and the Asp from GRGDSP peptide interact with the αIIB and the β3-subunit, respectively. The complex structure is stabilized by water molecules, and there is a salt bridge between R-216 from the β3-subunit and E-123 from the αIIB subunit near the contact site of the GRGDSP peptide.

The Arg from GRGDSP peptide interacts with the αIIB-subunit through bidentate hydrogen bonds with D-224 and a water molecule, as well as with F-160. It also has aromatic and hydrophobic interactions with Y-189, Y-190, F-231, and L-192. The first Gly of GRGDSP peptide interacts with RGD Y-190 from the αIIB-subunit, as well as A-218 from the β3-subunit. The Asp from GRGDSP peptide interacts with the β3-subunit through residues S-121, Y-122, S-123, N-215, and E-220 and a manganese ion. The residues SP from GRGDSP peptide interact with the β3-subunit through residues Y-122 and S-123, while Pro interacts with D-126, which is bound to another manganese ion (Zhu et al. 2013).

### Integrin αvβ3

Abnormal integrin αvβ3 expression is linked to cancer progression, tumor initiation, sustained tumor growth, distant metastasis, drug resistance development, and maintenance of stemness in cancer cells. The αvβ3-integrin is overexpressed in melanoma, breast cancer, and ovarian cancer. Several integrin αvβ3 antagonists have been developed, including RGD-4 C (ACDCRGDCFCG), a double cysteine-bridged peptide containing RGD, a PEGylated bicyclic single-cysteine bridging peptide, KCRGDCFC based on RGD-4 C, LXW7, LXZ2, IsoDGR, the isoDGR-based cyclopeptide CisoDGRC, linear small peptides RWrNM and RWrNK (containing d-arginine that can pass through the blood-brain tumor barrier), ProAgio (interacting outside the RGD binding site), and the RGD cyclic peptide cilengitide, the most clinically studied αvβ3 inhibitor molecule (Hynes 2002; Stupp et al. 2014; Gu et al. 2023)

The αvβ3 ligand-binding pocket contains three bivalent metal ion binding sites. The metal ion-dependent adhesion site directly coordinates the side chain of acidic residue characteristics of all integrin ligands, whereas the two external sites can also bind Mn^2+^, Mg^2+^, and Ca^2+^. This integrin has three basic conformations: bending (low-affinity ligand binding), medium affinity, and extended (high-affinity ligand binding). Integrin conformational changes are associated to divalent cations, activating antibodies (e.g., CBRLFA1/2), ligand-mimicking peptides, or intracellular activators (Van Agthoven et al. 2014; Xiao et al. 2004; Valdramidou et al. 2008; Zhu et al. 2013; Takagi et al. 2002; Nishida et al. 2006; Shattil et al. 2010; Ye et al. 2012; Schürpf and Springer 2011).

The Arg-Gly-Asp (RGD) sequence is the smallest integrin-binding motif that has been used for the development of numerous peptides and small molecules targeting integrins, such as αvβ3. These range from the linear tripeptide RGD (Kd = 89 nM) and the heptapeptide GRGDSPK (Kd = 12.2 nM) to cyclic RGD peptides with improved binding characteristics (more stable, effective, and specific than linear RGD peptides). Additionally, allosteric inhibitors of αvβ3 have also been developed, such as C19-9, a non-RGD inhibitor of αvβ3 with good affinity and excellent drug-like characteristics (Pierschbacher and Ruoslahti 1984; Indrevoll et al. 2006; Meyer et al. 2006; Sheldrake and Patterson 2014; Auzzas et al. 2010; Shimaoka and Springer 2003; Kapp et al. 2017; Pang et al. 2021, 2023; Gu et al. 2023).

Targeting both αvβ3 and α5β1 integrins is more effective in cancer treatment than targeting only one of them. For example, knottins 2.5D and 2.5F have nanomolar affinity for binding to αvβ3 (2.5D) or both αvβ3 and α5β1 (2.5F). The difference between 2.5D and 2.5F is four residues located aside of the RGD motif. The 2.5F and 2.5D binding to αvβ3 depends critically on the RGD loop flexibility (Van Agthoven et al. 2019; Kapp et al. 2017; Coller and Shattil 2008; Mason 2015; Caswell et al. 2008; Christoforides et al. 2012; Reynolds et al. 2009; Moore et al. 2013). Moreover, the human mab intetumumab (also known as CNTO 95) recognizes all αv-integrin family member (Kd = 210 ± 133 pM for αvβ3 and Kd = 250 ± 104 pM for αvβ5) (Trikha et al. 2004) and has anti-angiogenic and anti-tumor properties, inhibiting cell adhesion, migration, proliferation, and invasion. LM609, a mouse anti-human mab against αvβ3-integrin, has displayed significant anti-angiogenic activity in preclinical trials (Kobayashi et al. 2017; Almokadem and Belani 2012; Mitra et al. 2011; O'Day et al. 2011; Huang et al. 2013)

### Integrin αvβ6

The integrin αvβ6, which binds to vitronectin, tenascin-C, and fibronectin, plays a role in cell adhesion and migration. It is typically expressed at low levels or absent in healthy adult tissue epithelia but specifically upregulated during tissue repair, embryogenesis, and carcinogenesis. It also binds to latency-associated peptides (LAPs), resulting in the release of active TGF-β from the latent complex (Breuss et al. 1995; Busk et al. 1992; Dong et al. 2017; Larjava et al. 2011; Brzozowska and Deshmukh 2022; Urquiza et al. 2020).

High levels of αvβ6 expression in cancer patients with carcinomas of the skin, stomach, colon, breast, lung, oral mucosa, cervix, salivary gland, liver, ovary, and endometrium are usually associated with metastasis, tumor invasion, and a decrease in the median survival time of patients. High levels of αvβ6 expression combined with MMP-9, eIF4E, or Ets-1 are a prognostic indicator in patients with gastric cancer, colorectal cancer, and non-small cell lung cancer. Thus, integrin αvβ6 is a potential target for cancer treatment and diagnosis (Ahmed et al. 2002a, b; Berghoff et al. 2014; Koivisto et al. 2018; Lian et al. 2016; Niu et al. 2014; Niu and Li 2017; Peng et al. 2014; Wang and Hielscher 2017; Brzozowska and Deshmukh 2022). In fact, the peptide A20FMDV2 (NAVPNLRGDLQVLAQKVART), derived from the foot and mouth virus, exhibits high selectivity and affinity for the αvβ6 integrin, allowing αvβ6+ tumor detection using single-photon emission computed tomography (SPECT) and positron emission tomography (PET). This peptide, radiolabeled with 4-[18F] fluorobenzoic acid, is utilized in microPET for cancer imaging of αvβ6+ tumor cells (Brzozowska and Deshmukh 2022; Saleem et al. 2020; Hausner and Bold 2019).

The A20 peptide, incorporated into the DG loop of the HAdV-D10 fiber knob, enables the virus to selectively target and eradicate αvβ6+ tumor cells. Furthermore, the oncolytic adenovirus AdΔΔ modified with the A20FMDV2 selectively targets αvβ6 integrin and specifically eliminates αvβ6+ tumor cells. In fact, this virus is highly selective for αvβ6+ pancreatic cancer cells, which is particularly promising in treating metastasis of the incurable αvβ6+ pancreatic ductal adenocarcinoma (Bates et al. 2022; Man et al. 2018).

The αvβ6 integrin ligands have been used for designing chimeric antigen receptor (CAR) cells, to eradicate αvβ6+ cancer cells. The A20-2G and A20-4G CAR-constructs have been designed for targeting cholangiocarcinoma, a deadly form of bile duct cancer. These CARs contain peptide A20 fused with a second-generation CD28/CD3ζ signaling domain or with a fourth-generation CD28/4-1BB/CD27/CD3ζ signaling domain. Another CAR T cell construct, co-expressing CXCR2, the cognate IL-8 receptor, shows superior anti-tumor activity against αvβ6+ ovarian or pancreatic tumor xenografts. Furthermore, a CAR construct containing A20 peptide fused to CD28 + CD3 endodomain co-expressing an IL-4-responsive fusion gene (4αβ) is very potent in vivo in mice harboring αvβ6+ ovarian, breast, and pancreatic tumor xenografts. Conversely, a CAR construct has been built with a high-affinity 12-mer peptide (Bpep) specific for αvβ6 integrin and a human IgG4 hinge-Fc extracellular domain fused to the cytoplasmic tail of CD3-zeta. Primary human cytotoxic T lymphocytes expressing this CAR selectively eliminate αvβ6+ ovarian tumor cells. (Phanthaphol et al. 2021; Whilding et al. 2017, 2019; Uusi-Kerttula et al. 2018; Pameijer et al. 2007).

### Integrin αvβ8

The αvβ8 integrin binds specifically to the RGD peptide, present in vitronectin, collagen IV, and the main ligand latent TGFβ1/3. Initially, Itgb8 gene expression is detectable in most organs except for adipose tissue and blood. The αvβ8 integrin-mediated regulation of TGFβ signaling is crucial for the normal functions of immune cells and plays a role in the initiation and progression of various cancers. The reduced αvβ8 integrin expression correlates with higher-grade tumors in squamous cell carcinomas, whereas upregulation of β8 integrin expression in pancreatic cancer cells leads to more malignant disease and enhanced resistance to chemotherapy. Moreover, β8 integrin expression promotes perivascular growth of glioblastoma (GBM) cells and their invasion along blood vessels. GBM cell lines expressing low levels of integrin β8 are less invasive, while those expressing high levels of integrin β8 increase tumor growth and perivascular invasion. The αvβ8 integrin plays a pro-tumorigenic role in GBM stem cells, promoting tumor recurrence following radiation and chemotherapy; and there is a correlation between αvβ8 integrin expression and the severity of metastatic lesions in metastatic brain tumors (McCarty 2020; Reyes et al. 2013; Tchaicha et al. 2011; Lathia et al. 2015; Malric et al. 2019; Schittenhelm et al. 2013).

## Structural insights of peptide ligand molecular interaction with integrins αvβ3, αvβ6, and αvβ8

**Fig. 3:**
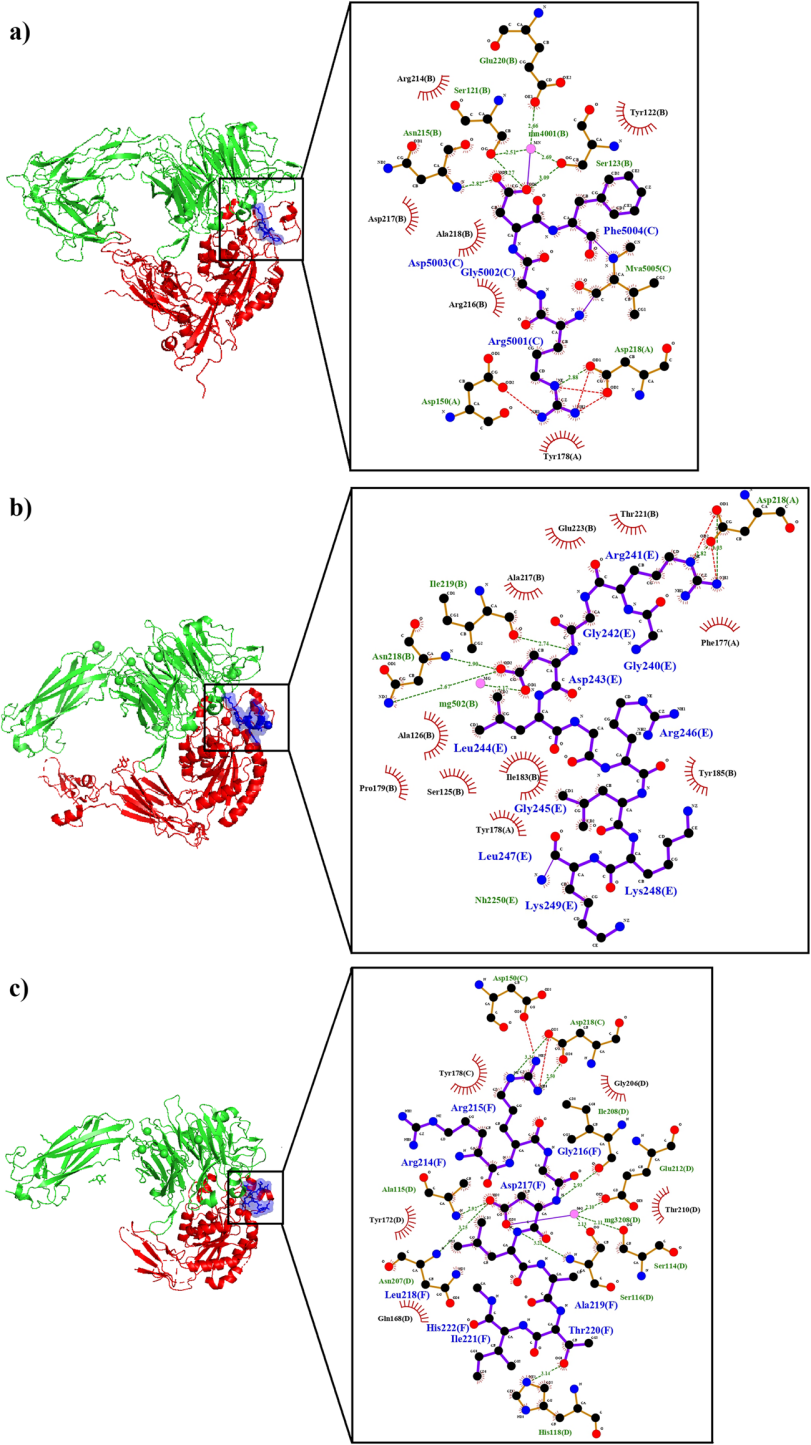
(**a**) Structure of the extracellular segment of integrin αvβ3 in complex with cyclic RGD-peptide (αv-subunit in green, β3-subunit in red, and RGD peptide in blue) with its interaction map of the RGD-peptide (C) with the α5-subunit (A) and β-3 subunit (B). (**b**) Structure of integrin αvβ6 in complex with latency-associated peptide (αv-subunit in green, β6-subunit in red, and latency-associated peptide in blue) with its interaction map of the RGD-peptide (E) with the α5-subunit (A) and β-3 subunit (B). (**c**) Structure of integrin αvβ8 in complex with pro TGF-β1 peptide (αv-subunit in green, β8-subunit in red, and pro TGF-β1 peptide in blue) with its interaction map of the RGD-peptide (F) with the α5-subunit (C) and β-3 subunit (D)

Numerous crystal structures of integrins αvβ3, αvβ6, and αvβ8 in complex with RGD-peptides have been obtained by X-ray diffraction (Xiong et al. 2002; Dong et al. 2014; Wang et al. 2019). Here, we analyze the crystal structures found in the protein data bank of integrin αvβ3 in complex with cyclic RGDF-peptide at a resolution of 3.20 Å (PDB: 1L5G) (Xiong et al. 2002), integrin αvβ6 in complex with GRGDLGRLKK-peptide at a resolution of 2.50 Å (PDB: 4UM9) (Dong et al. 2014), and integrin αvβ8 in complex with pro TGF-β1 peptide GRRGDLATIH at a resolution of 2.77 Å (PDB: 6OM2) (Wang et al. 2019). The Arg residue from the RGD motif, present in the peptides binding to these integrin, interacts with the α subunit, while the Asp from this motif interacts with the β subunit (Fig. 3).

The structures of the αv-subunit in the integrins αvβ3, αvβ6, and αvβ8 are very similar, with few changes in the position of main chain atoms. The peptide binding site on these integrins is also very similar. In fact, the R-residue of the RGD-motif contained in these peptides interacts with αv-subunit residues Y-178 and D-218 of these three integrins, D-150 with integrins αvβ3 and αvβ8, Q-180 with integrins αvβ6 and αvβ8, and F-177 with integrin αvβ8. The Arg from cyclic RGDF-peptide interacts with W-179 of the αv-subunit. However, changes in the position of several side chains of the αv-subunit, probably caused by interactions with the β-subunit, lead to changes in the αv-residues that interact with the R-residue of the RGD motif.

Additionally, the G-residue of the RGD-motif interacts with residues from the β-subunit in these three structures: R-216 and A-218 from the β3-subunit, T-221 and I-219 from the β6-subunit, and I-218 and T-210 from the β8-subunit. Meanwhile, the D-residue of the RGD-motif is buried and interacts with a pocket formed by residues S-121, N-215, R-216, E-220, and two manganese ions in the β3-subunit; by residues S-125, A-126, P-179, I-183, A-217, N-218, I-219, E-223, and one calcium and one magnesium ion in the β6-subunit; and with residues S-114, A-115, S-116, G-206, N-207, I-208, E-212, and one calcium and magnesium ion in the β8-subunit. The F residue of the RGDF cyclic peptide forms an aromatic interaction with Y-122 from the β3-subunit, which is not observed in the β6- and β8-subunits.

The first Arg of peptide GRRGDLAT interacts with Y-172, and Asp from RGD interacts with D-216 from β8-subunits. In addition, a hydrophobic pocket in the β6-subunit formed by an intramolecular disulfide bond between the C-180 and C-187 residues (also present in β3 and β8), and neighboring residues interact with L-214 and L-217 from the GRGDLGRLKK peptide. The molecular characteristics of this pocket vary among the three integrins, and according to experimental evidence, it is involved in the specificity of binding of these integrins. Furthermore, the structure indicates that the binding site for the C-terminus of the GRGDLGRLKK and GRRGDLATIH peptides is located in this pocket on the αvβ6 and αvβ8 integrins, respectively.

Also, the first Gly-residue of the GRRGDLAT peptide interacts with D-150 and Y-178 from the αv-subunit, while the first Arg of this peptide forms a hydrogen bond with Y-172 from the β8-subunit. The Leu-residue of the GRRGDLAT peptide interacts with A-115 from the β8-subunit, and the Thr-residue of the same peptide interacts with Ser-116 and His-118 from the β8-subunit (Wang et al. 2019).

A salt bridge is formed between the αv and β3 subunits through the interaction of an αv chain Asp and a β3 chain Lys, near the contact site of the RGD-peptide as reported by Xiong et al. (2002). Similarly, Dong et al. (2014) observed a salt bridge between the αv-subunit D-148 and β6-subunit K-170.

## Characteristics of the surface area buried on the integrin after peptide complex formation

In the five complexes analyzed here, the buried surface area of the peptides on the integrins ranges from 204.1 to 556.7 Å^2^, with mostly side chain atoms involved (between 77.2% and 96.1%), which is crucial for binding specificity to different integrins. Additionally, the integrin surface area buried is predominantly hydrophobic (between 60.7% and 73.4%), despite the main interaction being salt bridges involving the R and D residues in the peptide ligands (see also Table 2). Therefore, when designing an integrin-specific peptide or small molecule ligand, it is necessary to consider not only the RGD residues but also the surrounding residues, as they are responsible for the peptide’s specificity. Although the RGD residues are the main integrin binders, the surrounding peptide residues can cause steric hindrance, which may modify the specificity of peptide binding.

**Table 2:** Properties of the integrin peptide complex

Integrin complex	PDB	Sequence	Buried surface				MW*	Theoretical pI	GRAVY**
			Total (A^2^)	Apolar (%)	Backbone (%)	Side chain (%)			
αvβ6-RGDLXXL/I motif of TGF-β1	4UM9	GRGDLGRLKK	556.7	62.3	18.4	81.6	1099.30	11.00	-1.390
αvβ3-RGD peptide ligand	1L5G	RGDF	204.1	70.0	14.8	85.2	484.51	5.96	-1.400
αvβ8-proTGF-beta1 ligand peptide	6OM2	GRRGDLATIH	506.9	60.7	3.9	96.1	1095.23	9.61	-0.710
α5β1-RGD motif in fibronectin	4WK0	RGD	234.8	64.7	22.2	77.8	337.34	5.96	-2.800
αIIbβ3-RGD peptide complex	3ZE1	GRGDSP	331.3	73.4	22.8	77.2	587.59	5.84	-1.867

*Molecular weight. **Grand average of hydropathicity
